# The Popeye Domain Containing Genes and Their Function as cAMP Effector Proteins in Striated Muscle

**DOI:** 10.3390/jcdd5010018

**Published:** 2018-03-13

**Authors:** Thomas Brand

**Affiliations:** Cardiovascular Function, National Heart and Lung Institute, Imperial College London, Imperial Centre for Translational and Experimental Medicine, Rm. 337, Du Cane Road, London W12 0NN, UK; t.brand@imperial.ac.uk; Tel.: +44-207-594-8744

**Keywords:** Popeye domain, cAMP binding, effector protein, cardiac arrhythmia, limb-girdle muscular dystrophy, atrioventricular block

## Abstract

The Popeye domain containing (POPDC) genes encode transmembrane proteins, which are abundantly expressed in striated muscle cells. Hallmarks of the POPDC proteins are the presence of three transmembrane domains and the Popeye domain, which makes up a large part of the cytoplasmic portion of the protein and functions as a cAMP-binding domain. Interestingly, despite the prediction of structural similarity between the Popeye domain and other cAMP binding domains, at the protein sequence level they strongly differ from each other suggesting an independent evolutionary origin of POPDC proteins. Loss-of-function experiments in zebrafish and mouse established an important role of POPDC proteins for cardiac conduction and heart rate adaptation after stress. Loss-of function mutations in patients have been associated with limb-girdle muscular dystrophy and AV-block. These data suggest an important role of these proteins in the maintenance of structure and function of striated muscle cells.

## 1. Introduction

**Figure 1:**
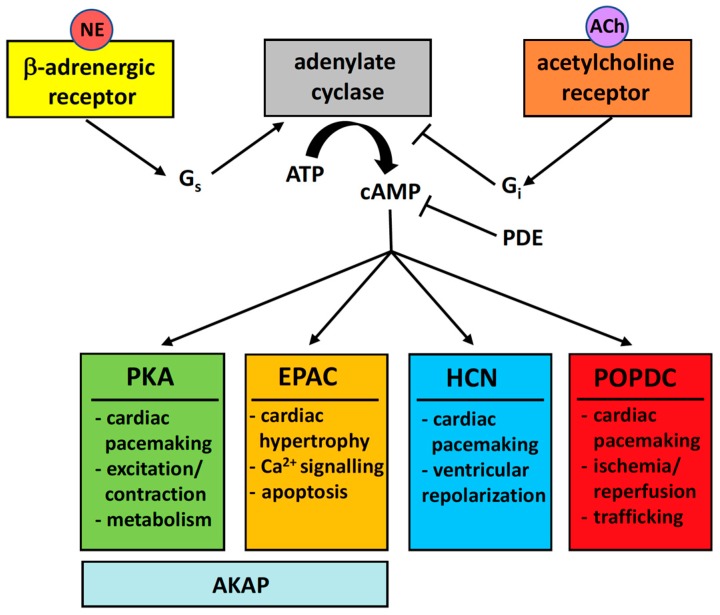
Four cAMP effector proteins are expressed in the heart. Norepinephrine (NE) secreted by sympathetic neurons binds to the β-adrenergic receptor leading to G_s_ activation and synthesis of cAMP by adenylate cyclase (AC). Acetylcholine (ACh) is secreted by parasympathetic neurons and binds to muscarinergic ACh receptors leading to an activation of G_i_ causing AC inhibition. The balance of sympathetic and parasympathetic input therefore determines the level of cAMP production. cAMP production in cells is compartmentalized and this is mainly achieved by phoshodiesterases (PDE), which limits cAMP diffusion through degradation. Four effector proteins sense cAMP levels. The best-characterized effector protein is protein kinase A (PKA), which plays a role in cardiac pacemaking, excitation/contraction coupling and cardiac metabolism. The exchange factor directly activated by cAMP (EPAC) has been linked to cardiac hypertrophy, Ca^2+^-signaling and apoptosis. Often, PKA and EPAC are bound by the same anchor protein (AKAP) along with protein substrates and other enzymes. The hyperpolarization-activated cyclic nucleotide-gated (HCN) channels are important for cardiac pacemaking and ventricular repolarization. Finally, the Popeye domain containing (POPDC) proteins are the most recently identified class of effector proteins and important for cardiac pacemaking, the survival of cardiac myocytes after ischemia/reperfusion and membrane trafficking.

The second messenger cyclic adenosine 3′,5′-monophosphate (cAMP) activates an evolutionary ancient and universally important intracellular signaling pathway. Several effector proteins have been identified, which bind cAMP with high affinity causing their activation and leading to the execution of a variety of biological activities (Figure 1).

The most important and prominent cAMP effector protein is protein kinase A (PKA), which upon binding of up to four cAMP molecules to the regulatory subunit causes an activation of the catalytic subunits leading to the phosphorylation of many protein substrates. It is estimated that around 350 proteins are phosphorylated in response to β1-adrenergic stimulation, many of which are targeted by PKA [1]. PKA protein substrates are diverse but include proteins regulating energy metabolism, cardiac pacemaking and excitation/contraction coupling. PKA is bound by a number of scaffolding proteins called A kinase anchoring proteins (AKAPs) [2]. In cardiac myocytes, several AKAPs are expressed including D-AKAP1, D-AKAP2, AKAP15/18, AKAP79/150, Yotiao, mAKAPβ, AKAP-Lbc, Gravin and SKIP [3]. AKAPs are responsible to target PKA to specific subcellular domains such as the plasma membrane, transverse (T)-tubules, sarcoplasmatic reticulum, myofilaments and nuclear envelope [2]. AKAP proteins not only bind PKA but also protein substrates and other signaling molecules [2]. Recent data suggest that at physiological cAMP concentrations, dissociation of the PKA holoenzyme does not occur [4]. PKA being bound by different AKAPs in the cell is therefore only able to phosphorylate substrates in its direct vicinity, which enhances specificity and compartmentation of the cellular response.

EPAC (exchange factor directly activated by cAMP) is a guanine-nucleotide exchange factors for the Ras-like GTPases, Rap1 and Rap2 [5]. Two EPAC isoforms, EPAC1 and EPAC2 exist in mammals and are thought to modulate Ca^2+^-homeostasis and hypertrophy in cardiac myocytes [6]. Interestingly, EPAC1 and EPAC2 differ regarding their subcellular localization. EPAC2 is mostly present at the T-tubules and EPAC1 is localized perinuclear [7,8]. EPAC1 is also present in mitochondria and loss of EPAC1 reduces infarct size and cardiomyocyte apoptosis induced by myocardial ischemia/reperfusion injury [9]. EPAC also has pro-arrhythmic effects probably due its ability to decrease potassium currents [10]. Chronic EPAC activation leads to cardiac hypertrophy [11].

Another important effector protein in cardiac myocytes is HCN4, which is a member of the family of hyperpolarization-activated cyclic nucleotide gated channels (HCN). HCN genes encode nonselective voltage-gated cation channels, which are abundantly expressed in cardiac pacemaker cells in the sinoatrial (SAN) and atrioventricular nodes (AVN) but are also present in parts of the ventricular conduction system [12]. Upon binding of cAMP, the HCN4 channel opens more rapidly and completely [13]. This property has led to the assumption that the cAMP-mediated enhancement of HCN channel activity is largely responsible for the increase in heart rate in response to β-adrenergic stimulation. However, the importance of HCN4 in cardiac pacemaking remains controversial [14,15]. Heart rate adaptation in response to adrenergic stimulation remains intact after SAN-specific ablation of the *Hcn4* locus in mice [16]. Moreover, heart rate acceleration in response to adrenergic signaling is unaffected in patients carrying a cAMP-binding site mutation in *HCN4* [17]. To accommodate these facts, it has been proposed that HCN4 may act as a depolarization reserve and its main role may be to counteract parasympathetic slowing of the heart rate [18,19]. In addition to its importance in cardiac pacemaking, HCN channels apparently also have a role in impulse propagation in the SAN and are also essential for ventricular repolarization [20,21]. Importantly, in cardiac hypertrophy, both HCN2 and HCN4 are induced in the ventricular working myocardium causing a prolongation of the cardiac action potential and probably being responsible for the development of cardiac arrhythmia in the hypertrophied ventricle [22].

Although the Popeye domain containing genes were isolated around the same time as EPAC genes [23,24,25,26], it took more than a decade to realize that POPDC proteins constitute a novel class of cAMP-effector proteins [27] and an additional four years to demonstrate that cAMP binding is essential for its function [28]. Here we will review the multiple roles of the POPDC gene family in striated muscle biology and disease.

## 2. The Popeye Gene Family

The POPDC gene family consists of three genes namely *POPDC1* (also known as *BVES*), *POPDC2* and *POPDC3*. In vertebrates, *POPDC1* and *POPDC3* are tandem-organized and in the human genome are present on chromosome 6q21, while *POPDC2* is located on chromosome 3q13.33. [26]. *POPDC1* is the most complex gene with 8 exons, while *POPDC2* and *POPDC3* have only 4 exons. *POPDC1* probably represent the ancestral gene and the other two paralogs were generated through two gene duplication events. The first duplication likely took place at the base of chordate evolution [29]. In support of this view, basic chordates (*Tunicates*, *Cephalochordates*) have two tandem-organized POPDC genes with homologies to *POPDC1* and *POPDC3. POPDC2*, which is only found in vertebrates, was probably generated by a second gene duplication event, which probably took place at the base of vertebrate evolution. This view is supported by the fact that *POPDC2* is only present in vertebrates. Moreover, *POPDC2* and *POPDC3* display a similar gene structure and higher similarity at the protein level [30]. The POPDC gene family also evolved independently in invertebrates, where gene duplication events also took place. While molluscs, annelids and dipterans such as *Drosophila* have only a single POPDC gene; many insects have two POPDC genes and some invertebrates (for example the water flea) have multiple POPDC genes. Interestingly, there are also invertebrate species, which lost their POPDC gene during evolution and a prominent example for this is *Caenorhabditis elegans*. Thus, for some species, a lack of POPDC genes is fully compatible with life. We can trace the presence of POPDC genes down to Hydra and the gene family is also found in some protozoan species. We can therefore conclude that POPDC genes have evolved at the base of the animal kingdom. No POPDC genes are found in plants or fungi. Interestingly, the cAMP-binding domain (CNBD) of the bacterial catabolite activator proteins (CAP), also known as catabolite receptor protein (CRP), which are involved in transcriptional regulation of metabolism display the highest similarity of a non-POPDC protein to the Popeye domain.

## 3. POPDC Proteins

**Figure 2:**
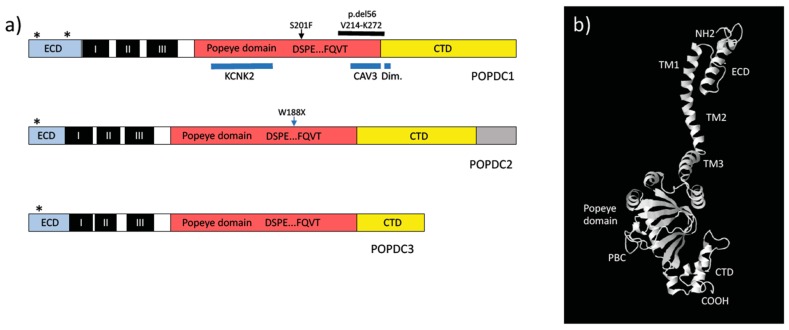
Structure of the Popeye domain containing proteins. (**a**) Schematic structure of the human POPDC isoforms. The three POPDC proteins share a similar protein structure. In each case a 20–40 amino acid long extracellular domain (ECD), which harbors one or two N-glycosylation sites (asterisks) is followed by three transmembrane (TM) domains (I–III, black boxes). The cytoplasmic portion of the POPDC proteins consists of the Popeye domain (red box) and the carboxyterminal domain (CTD, yellow box). The phosphate-binding cassette (PBC), which is thought to bind cAMP, consists of the two tetrapeptides DSPE and FQVT. In case of POPDC1, the locations for the KCNK2 (TREK-1) [28] and CAV3 [33] binding sites and the putative dimerization motif [34] are indicated (blue bars). POPDC2 generates three alternative splice products, which differ in their carboxy-terminus labeled by a grey box. Mutations in POPDC1 and POPDC2, which have been identified in patients with muscle and heart disease are indicated above each protein model. (**b**) 3-D structure of POPDC3. A homology-based structural model was generated with the help of the Phyre 2 algorithm [35]. The resulting structure was visualized with the help of First Glance in JMOL [36].

The POPDC proteins are medium-sized transmembrane proteins. They consist of a short extracellular domain (ECD, Figure 2), which in case of POPDC1 has been shown to be subject to N-glycosylation. Potentially, N-glycosylation is more extensive in skeletal muscle and brain than in the heart [31,32]. A consensus sequence for N-glycosylation is also present in the ECD of POPDC2 and POPDC3. POPDC proteins all have three transmembrane domains. In the cytoplasmic part, the conserved Popeye domain is present, which functions as a cAMP-binding domain [27].

The Popeye domain is slightly larger than a typical cyclic nucleotide monophosphate (cNMP)-binding domain (CNBD), suggesting that additional functions other than cAMP-binding reside in the Popeye domain. Indeed, the binding sites of KCNK2 (TREK-1) or CAV3 have been mapped to the Popeye domain of POPDC1 [28,33] (Figure 2). Soon after its discovery, the dimerization of POPDC1 was first described [31,32]. Dimerization is stabilized through disulfide bridge formation. In order to map the sequences, which mediate dimerization, a series of experiments including carboxy-terminal deletions, peptide-mapping and site-directed mutagenesis was utilized [34]. Dimerization appeared to depend on two conserved lysine residues at the end of the Popeye domain. Mutation of the dimerization motif interfered with cell adhesion, and epithelia formation. There are probably other sequences in POPDC1 also responsible for dimerization as POPDC1 protein, which lacked these sequences, were still able to homodimerize [37].

## 4. The Popeye Domain Is a Novel cAMP-Binding Domain

**Figure 3:**
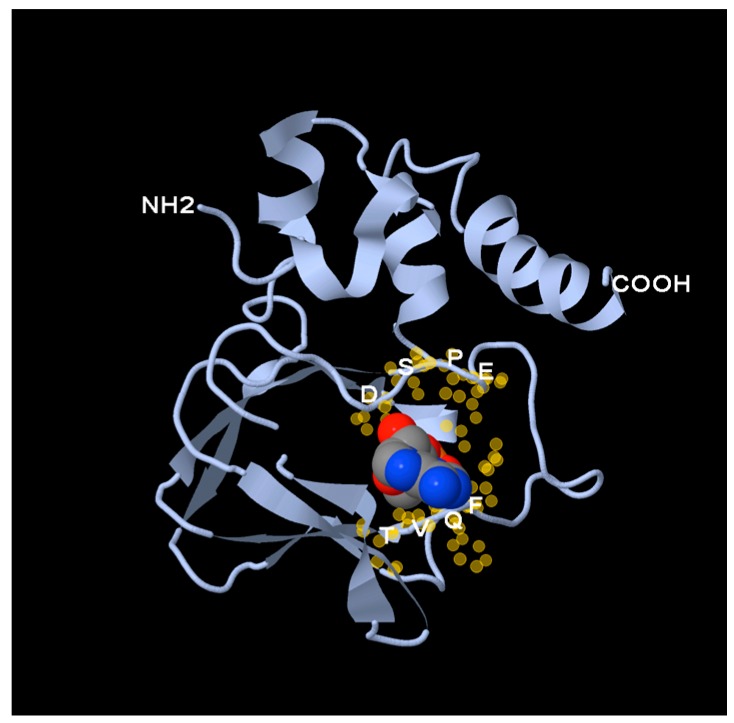
Structure of the Popeye domain. A homology-based structural model of human POPDC1 was generated. A space-filling model of cAMP was placed in the putative phosphate-binding cassette. The DSPE and FQVT motifs, depicted as yellow halos, surround the cAMP molecule and as mutagenesis studies suggest, seem to be directly involved in cAMP-binding.

It is thought that the main function of the 150 amino acids long Popeye domain is to bind cAMP [27]. The structural prediction of the Popeye domain revealed a similarity to the CNBD of the catalytic subunit of PKA [27]. The structure of the CNBD is categorized as jelly-roll β-barrel fold, which is found in many proteins. However, not all of these proteins bind cyclic nucleotides but other ligands [38]. A conserved feature of the CNBD is the phosphate-binding cassette (PBC), which directly makes contact to cAMP and consists of a short α-helix and a loop located between β-sheets 6 and 7. Two conserved residues found in all PBCs include an arginine, which binds to the phosphate group of cAMP and a glutamate that binds the 2′-OH group of the ribose [39]. Those proteins that share the jelly-roll β-barrel fold structure but lack these arginine and glutamate residues, bind to ligands other than cNMPs. It is, therefore, essential that sufficient experimental evidence will be generated that it is unequivocally established that POPDC proteins bind cAMP despite their lack of a canonical PBC. The non-canonical PBC of the Popeye domain is thought to consist of two conserved sequence motifs (FL/IDSPEW/F) and FQVT/S), which are linked by a non-conserved sequence of variable length [27] (Figure 3).

Several of these evolutionary conserved residues were mutagenized by a charge-to-alanine strategy in POPDC1 and POPDC2 and as expected, the resulting mutant proteins displayed a reduction in the cAMP-binding affinity supporting their involvement in nucleotide-binding [27].

Assays were developed to demonstrate cAMP-binding by POPDC proteins. The first one, is a cAMP agarose precipitation assay, which established cAMP-binding of native POPDC1 protein extracted from the chicken heart [27]. POPDC1 was also precipitated by cGMP agarose and free cGMP was able to elute POPDC1 from cAMP agarose and thus, at this stage it was unclear, whether POPDC1 binds cAMP selectively, or both cyclic nucleotides with equal affinity. The agarose-precipitation assay also established that the three POPDC proteins are all able to bind cAMP [27].

A recombinant POPDC1 protein encompassing the cytoplasmic part of the protein was employed in a competitive radio-ligand binding assay, which established an IC_50_ concentration of 118.4 ± 7.1 nM for cAMP and of 5.27 ± 0.68 μM for cGMP [27]. The approximately 40-fold difference in affinity between cAMP and cGMP is typical for a cAMP-binding protein and similar differences in affinities for both nucleotides have been described for the CNBDs of PKA [40].

A third assay demonstrating cAMP binding was developed based on the interaction of POPDC1 with the two-pore potassium channel KCNK2 (TREK-1) [27]. A bimolecular fluorescence resonance energy transfer (FRET) assay using CFP-tagged POPDC1 and YFP-tagged TREK-1 was utilized. Addition of the β-adrenergic agonist isoproterenol or direct stimulation of adenylate cyclase by forskolin caused a rapid decline of the YFP/CFP ratio with a kinetic, typical for an adenylate cyclase-dependent process [27]. A mutant protein, carrying a mutation in D200, which is one of the residues of the DSPE motif in the PBC of POPDC1 (Figure 3), did not cause any changes in the FRET signal after the addition of isoproterenol. Likewise, nitroprussid, which raises cGMP levels in cells, did not affect the FRET signal, which supports the notion that at physiological concentration levels, POPDC1 protein binds cAMP but not cGMP.

Finally, evidence for cAMP-binding of POPDC proteins stems from the discovery of a POPDC1^S201F^ mutation, which is present in patients, which develop a limb-girdle muscular dystrophy (LGMD) phenotype [28]. The serine residue 201 of POPDC1 is part of the PBC and change of serine into phenylalanine is likely to reduce the cAMP-binding affinity. Indeed, measurements of cAMP affinity established a loss of about 50% in case of the mutant protein [28]. Thus, there is ample of experimental and genetic evidence, which establishes the Popeye domain as a novel cAMP-binding domain with a highly divergent protein sequence [28]. However, so far, the molecular characterization mostly dealt with POPDC1 protein and therefore it will be important to extend these experiments also to the other two family members and to determine, whether cyclic nucleotide specificity is the same for each of the POPDC proteins, and whether all three POPDC isoforms bind cAMP with equal affinity.

## 5. Expression Pattern of POPDC Genes

**Figure 4:**
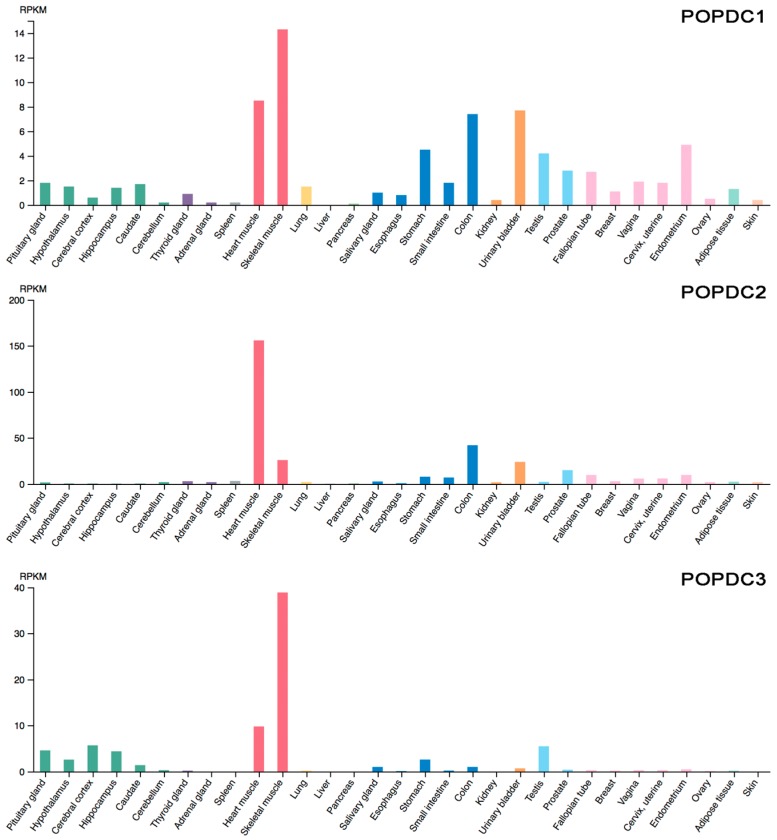
Expression of POPDC genes in the human body. RNA seq data of *POPDC1*, *POPDC2* and *POPDC3* in human tissue. RNA expression is quantified as reads per kilogram per million mapped reads (RPKM). Data were copied from the Human Protein Atlas website [46,47,48].

RNA expression data for the POPDC family members revealed the highest expression level of POPDC genes in striated muscle tissue (Figure 4) [26]. While *POPDC2* is preferentially expressed in the heart, *POPDC1* and *POPDC3* show higher expression levels in skeletal muscle. Of the three genes, *POPDC1* shows a more widespread expression than *POPDC2* and *POPDC3.* In many tissues, *POPDC1* and at least one other POPDC gene is co-expressed. Apart from striated muscle, expression was also observed in smooth muscle cells, neurons, and epithelial cells [41]. Conflicting data have been published for the heart regarding the presence of POPDC1 in non-muscle cell types of the heart. Several studies from my lab revealed an exclusive expression in cardiac myocytes [41,42,43], while another group reported expression in cardiac myocytes but also presence of POPDC1 in the epicardium and in coronary arteries [25,44,45]. The LacZ reporter gene, which was knocked into *POPDC1* did not reveal a significant expression in any of the non-muscle cell lineages in the adult heart [26,41]. However, a shortcoming of these data is the fact that the LacZ reporter gene may not fully recapitulate the expression domains of the endogenous gene. On the other end, it is equally conceivable that the antibodies employed may be cross-reactive. A thorough investigation of the expression patterns of POPDC genes and proteins in vertebrate embryos and in the adult seems to be required to settle this case.

**Figure 5:**
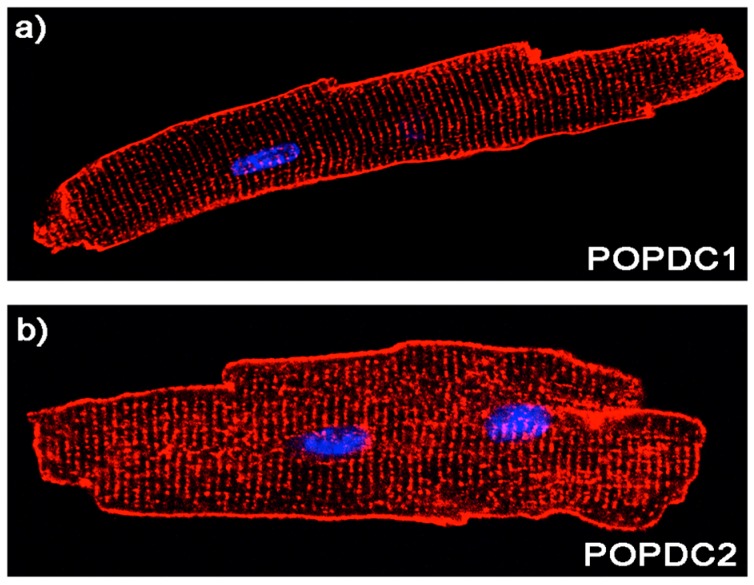
Expression of POPDC1 and POPDC2 in cardiac myocytes. Isolated adult cardiac myocytes were immunostained with (**a**) POPDC1 and (**b**) POPDC2 antibodies, respectively.

Immunolocalization of POPDC proteins revealed an expression at the plasma membrane of isolated cardiac myocytes and a similar pattern was observed in cardiac tissues. POPDC proteins have been described to be present at the lateral membrane, intercalated disks, costameres [33], t-tubules and caveolae [33] (Figure 5). In addition, POPDC1 was also localized to the nuclear envelope [49], and in the nucleoplasm [50]. Plasma membrane localization was also reported for skeletal muscle, however no detailed analysis of the subcellular localization in different membrane compartments has been reported [28]. A recent report, which was looking for novel intercalated disk proteins described a preferential localization of POPDC2 in the intercalated disk of ventricular myocytes of the human and canine heart, arguing for an important function of POPDC2 for electrical cell-to-cell coupling, and impulse propagation [51].

## 6. Animal Models to Define the Function POPDC Genes

In order to define the function of POPDC genes, the *Popdc1* and *Popdc2* genes were ablated in mice by substituting the first coding exon with a LacZ reporter gene [27,52] (Table 1). The resulting homozygous mutants were viable and no embryonic lethality has been reported. Phenotype analysis was guided by the LacZ expression pattern [41]. In the adult mouse heart, *Popdc1* and *Popdc2* are expressed in cardiac myocytes, while no expression in smooth muscle or non-muscle cells is observed [27,41]. *Popdc1* displays higher level of expression in atria than in ventricles, while *Popdc2* is evenly expressed in both chamber types [27]. Both genes display a strong expression in the sinoatrial (SAN) and atrioventricular nodes (AVN) as well as in the ventricular conduction system [27]. The high-level expression of POPDC1 and 2 genes in the cardiac conduction system prompted the investigation of the functional status of it in the null mutant animals. While both *Popdc1* and *Popdc2* null mutants displayed normal heart rhythm at rest, a stress-induced sinus bradycardia developed in response to physical or emotional stress and after β-adrenergic receptor stimulation [27]. ECG analysis revealed the presence of stress-induced sinus pauses. The length of each sinus pause was variable and could be brief or more prolonged. Interestingly, the arrhythmia phenotype developed in both null mutants in an age-dependent manner and was absent in young adults and gradually worsened with increasing age [27]. The phenotype was also present in isolated Langendorff-perfused hearts and a normal cholinergic response was observed after carbachol treatment, which suggests a primary defect in the cardiac pacemaker.

Structural analysis of the sinoatrial node (SAN) morphology revealed significant structural alterations. The SAN is located at the border between vena cava and right atrium and consists of nodal myocytes, which also called spider and spindle cells due to their irregular shape and the long neurite-like cellular extensions [56]. Nodal myocytes are embedded in a rich extracellular matrix and are poorly electrically coupled. In both, *Popdc1* and *Popdc2* null mutants, pacemaker cells displayed a reduced number of cellular extensions, and overall the SAN had a more compact structure. Moreover, a reduction of nodal myocardium was noted in the inferior part of the SAN [27].

The molecular basis of the sinus node dysfunction in *Popdc1* and *Popdc2* null mutants is not well understood. An attractive hypothesis involves a modulatory role of POPDC proteins for the pacemaker current I_f_. Therefore, I_f_ current density and activation time was measured in isolated SAN myocytes from WT and *Popdc2* null mutant mice [27]. Cells of both genotypes were indistinguishable in current density under basal conditions and after superfusion with 8-Br-cAMP. While I_f_ was not investigated in the *Popdc1* null mutant, therefore an interaction of POPDC1 and HCN4 cannot be excluded, however, the similarity of phenotypes of both *Popdc1* and *Popdc2* null mutants makes it very unlikely that I_f_ function is modulated by POPDC1.

One of the first POPDC-interacting protein identified in the heart was the potassium channel TWIK-related K^+^ channel 1 (TREK-1) or KCNK2 [27]. This ion channel is a member of the two-pore domain potassium channel (K_2_P) family. TREK-1 gating is affected by a large number of stimuli including stretch, pH, temperature, phosphorylation, and is also modulated through the interaction with other proteins [57]. In the presence of POPDC1, TREK-1 produces a twofold higher current in *Xenopus* oocytes [27]. The effect is also observed when POPDC2 or POPDC3 were co-expressed. The doubling of TREK-1 current is probably due to an increase in membrane trafficking as more TREK-1 protein is detected at the cell surface when POPDC proteins are present. The effect of POPDC proteins on TREK-1 is sensitive to cAMP and is lost when cAMP levels were increased after applying the general PDE inhibitor theophylline or 8-Br-cAMP [27,28]. The interaction of POPDC proteins with TREK-1 has been mapped to the Popeye domain by deletion analysis (Figure 2). Based on the interaction of POPDC1 and TREK-1, a bi-molecular Förster-resonance energy transfer (FRET) sensor was constructed. The FRET ratio obtained at baseline decreased after the addition of isoproterenol or forskolin, suggesting that cyclic nucleotide-binding affects the interaction of POPDC1 with TREK-1 [27]. A cardiac specific knockout of *Kcnk2*, which encodes TREK-1, displays a stress-induced sinus bradycardia similar to the one observed in *Popdc1* and *Popdc2* null mutants [58], suggesting that the sinus bradycardia in POPDC mutants may in part be due to an impaired TREK-1 current.

However, it is likely that additional proteins may be involved. Cardiac pacemaking is governed by two oscillatory mechanisms, the membrane clock and the Ca^2+^-clock [59]. The membrane clock initiates depolarization in response to I_f_ but also involves the voltage-gated Ca^2+^-channels. The Ca^2+^-clock on the other hand is active in late diastole when Ca^2+^ is released from the sarcoplasmatic reticulum by the ryanodine receptors (RyRs) and exchanged for Na^+^ by NCX, which generates a depolarizing I_NCX_ current. Both clocks are coupled by Ca^2+^, which enters the cell through the L-type Ca^2+^ channels, which leads to a replenishment of the SR allowing the next pacemaker cycle to be initiated. Interestingly, it was recently reported that the sodium calcium exchanger NCX1 is a POPDC2-interacting protein [60] and the loss of NCX1 causes SAN dysfunction similar to the one observed in POPDC mutants [61]. It seems warranted to thoroughly investigate protein-protein interaction, subcellular localization, and function of proteins of the membrane and Ca^2+^ clocks in the different POPDC mutants.

The impact of ischemia/reperfusion injury was studied in the *Popdc1* null mutant [33]. Langendorff-perfused mutant hearts displayed a significantly lower functional recovery, while infarct size was larger. Isolated cardiac myocytes from *Popdc1* null mutants displayed altered Ca^2+^-transients and increased vulnerability to oxidative stress. Apparently *Popdc1* is involved in myocyte survival through the suppression of the pro-apoptotic Bcl2 interacting protein 3 (*Bnip3*) gene [62]. While skeletal muscle is not as well studied than cardiac muscle in POPDC null mutants, it has been shown that muscle regeneration is impaired in *Popdc1* null mutants, however, the underlying molecular mechanism has yet to be elucidated [63].

In *Popdc1* null mutant mice, the caveolar compartment is affected and caveolae were fewer in number, however their size was increased [33]. POPDC1 interacts with CAV3 through a consensus sequence present at the end of the Popeye domain (Figure 2). This could have profound effects on cardiac signal transduction given that caveolae in cardiac myocytes are clustering membrane receptors, signaling molecules, protein pumps and ion channels [64]. It has been reported that POPDC2 in human and canine cardiac myocytes are predominantly present at the intercalated disk [51]. The intercalated disk controls electrical coupling between cardiac myocytes and therefore is a major determinant of cardiac conduction. It will be interesting to study the structure and function of the intercalated disk in POPDC2 null mutants and to measure electrical conduction in *Popdc2* null mutants.

Loss-of function experiments were also conducted in zebrafish and morpholino-mediated knockdown of *popdc1* and *popdc2* caused cardiac arrhythmia and muscular dystrophy phenotypes [28,53] (Table 1). The cardiac arrhythmia was already present in the embryo and the severity of the phenotype increased in an age-dependent manner. In young larvae (4 days post-fertilization, dpf) typically a type I AV-block was present. At increasing age total heart block or even non-contracting hearts were also observed. The skeletal muscle phenotype in *popdc1* and *popdc2* morphants was characterized by an impaired formation of the myotendinous junction which probably caused the myofiber rupture, which was seen in both morphants [28,53]. The heart of many *popdc1* and *popdc2* morphants displayed a pericardial effusion, which is thought to indicate myocardial pumping deficiency. However, in the case of *popdc1* morphants, the pericardial effusion could also be based on a defective barrier formation of the skin due to impaired tight junction formation [65]. The molecular basis for the tight junction defect is probably due to abnormal accumulation of tight junction proteins, which is caused by an aberrant localization of atypical protein kinase C (aPKC) [65]. Interestingly, it has been demonstrated that another protein involved in cell contact formation, zonula occludens 1 (ZO1) is a POPDC1-interacting protein [66].

The contact structures between skeletal muscle fibers in the zebrafish tail musculature is formed by the myoseptum or myotendinous junction (MTJ) [67]. The MTJ is a complex structure, which is established by several plasma membrane proteins and various matrix proteins [68,69]. In the *popdc1* and *popdc2* morphants and in the *popdc1*^S191F^ mutant, the MTJ is abnormal in structure and in the *popdc1*^S191F^ mutant lacks the collagen matrix, which probably is essential for the MTJ to withstand the tensile forces of the contracting myofibers. Consistent with this hypothesis, large numbers of hypercontracted myofibers that lost contact to the MTJ are seen in *popdc1* and *popdc2* morphants and the *popdc1*^S191F^ mutant [28,53].

## 7. Evidence for a Role of POPDC Genes in Human Heart and Skeletal Muscle

The first mutation of *POPDC1* discovered in patients is the recessive *POPDC1^S201F^* mutation, which was reported to be associated with cardiac arrhythmia and muscular dystrophy [28]. This mutation alters serine 201, which is one of the invariant amino acids present in the putative cAMP binding domain (DSPE motif) (Figure 2, Table 1). The serine residue is substituted by phenylalanine, which may interfere with the cyclic nucleotide gaining access to the PBC. Measurement of the cAMP binding affinity revealed a 50% reduction in case of the mutant protein [28]. Moreover, the S201F mutant caused an increased level of TREK-1 current compared to wildtype (WT) while membrane transport of the channel protein was decreased [28]. This paradoxical finding of decreased membrane transport and increased current may be explained on the basis that POPDC1 in a complex with TREK-1 protein might be protected from getting inactivated by PKA-dependent phosphorylation [70]. Forced expression of POPDC1^S201F^ in HL1 cells affected the action potential [28]. Three patients from a 3-generation family of Albanian descent carried the POPDC^S201F^ mutation to homozygosity and developed an early onset second-degree AV-block and a late onset limb-girdle muscular dystrophy (LGMD2X, OMIM: #61812) [28]. Serum creatine kinase levels were elevated, and muscle biopsies showed dystrophic changes with increased fiber size variability, increased central nuclei, and a few necrotic fibers. Electron microscopic analysis of skeletal muscle biopsies of one of the patients carrying a POPDC1^S201F^ mutation revealed the presence of membrane discontinuities [28]. Similar discontinuities have been observed in patients with mutations in anoctamine-5 (*ANO5*). While only found in a single patient, these discontinuities are possibly an indication for impaired repair of the muscle plasma membrane [71]. Interestingly, another protein involved in membrane repair, dysferlin, has also been shown to be an interaction partner of POPDC1 [28]. Expression of the *popdc1* disease-associated mutation (S191F) in zebrafish caused similar phenotypes as in the patients. Skeletal muscle from homozygous *popdc1*^S191F^ mutants showed myofibrillar misalignment, aberrant formation of the myotendinous junction, myofiber detachment, and decreased membrane localization of POPDC1 and POPDC2 [28]. Electron microscopy revealed an absence of extracellular matrix at the myotendinous junction. Abnormalities of the zebrafish *popdc1^S191^* mutant heart also resembled the patient’s phenotype displaying an overall reduction in heart rate and stroke volume. Isoproterenol caused an increase in the number of embryos displaying a 2:1 AV block.

Recently, another recessive mutation in *POPDC1* was reported in a consanguineous family of Algerian origin [54]. The splice site mutation (c.816 + 2T > C) affects the splice donor site in intron 8 and may cause skipping of exon 8 resulting in a mutant protein with a predicted loss of 56 amino acids (POPDC1^del56 V217-K272^), (Figure 2, Table 1). Two siblings are affected, and both displayed a first-degree atrioventricular (AV)-block, high serum creatine kinase levels and evidence for a mild limb-girdle muscular dystrophy. Immunostaining of biopsies revealed a strong reduction in membrane staining of both, the mutant POPDC1 protein and also of POPDC2. Thus, despite POPDC1^S201F^ and POPDC1^del56 V217-K272^ mutations are at different position in the protein they induce a similar pathology and display impaired membrane trafficking [28,54]. The underlying molecular defects require further characterization.

*POPDC1* may also contribute to phenotype variability in other LGMDs. Interestingly, heterozygous *POPDC1* mutations were found in patients that also carried Lamin A (*LMNA*) or dysferlin (*DYSF)* mutations. This putative genetic interaction is probably of significance as both proteins, dysferlin and Lamin A also interact at the protein level with POPDC1.

Patients carrying a recessive *POPDC2* mutation were recently discovered [55]. This mutation (c.563G>A, p.Trp188Stop, POPDC^W188X^) resulted in the insertion of a premature stop codon at position 188 of *POPDC2*, leading in the protein to a deletion within the putative cAMP binding domain. Even though the FQVT motif of the PBC of POPDC2 was deleted in this mutant, cAMP responsiveness remained unaltered. Likewise, the interaction and modulation of the TREK-1 current remained unaltered. Therefore, currently the pathogenic mechanism is not fully clear. The deletion of part of the Popeye domain and the carboxyterminal domain may influence the kinetics of cAMP binding or could interfere with protein-protein interaction.

## 8. Conclusions

We recently proposed four working models of how POPDC proteins might modulate proteins in order to mediate cAMP signaling in striated muscle cells [68]. The first model, which is called the *switch model*, proposes that binding of cAMP to POPDC proteins causes an allosteric effect as it was observed for PKA, EPAC and HCN4 [72].

The *switch model* assumes that allostery not only affects POPDC proteins but also interacting proteins. Direct evidence for such a behavior is currently missing. Nonetheless, the TREK-1-POPDC1 FRET assay gives support for the presence of an allosteric effect. Experimental support is present for the *cargo model*, which proposes that POPDC acts on proteins by modulating their membrane trafficking. Strong support for this model comes from the observation that the POPDC1^S201F^ mutant not only affects membrane localization of the mutant protein but also of POPDC2. Similarly, membrane localization of TREK-1 in *Xenopus oocytes* is modulated by POPDC1 and is cAMP-sensitive.

The *shielding model* is a variation of the *switch model* taking into account that POPDC proteins and also some of the interacting proteins are getting phosphorylated in response to βAR activation [1]. It can be envisioned that cAMP binding and phosphorylation of POPDC proteins may lead to conformational changes that may also affect access of PKA or other kinases to their substrates.

Finally, the *sponge model* takes into account that POPDC proteins are abundant in cardiac and skeletal muscle cells and display a complex subcellular localization. POPDC proteins have a high affinity binding site for cAMP, which suggests that these proteins may have a strong impact on cAMP compartmentalization. POPDC proteins may assist adenylate cyclases, phosphodiesterases, and AKAP proteins to create nanodomains of cAMP signaling. Further work is required to confirm or refute these working models.

## Figures and Tables

**Table 1:** Cardiac and skeletal muscle phenotypes in model organism and patients.

Species	Mutation	Heart	Skeletal Muscle	References
**mouse**	*Popdc1*^-/-^	stress-induced sinus bradycardia	regeneration defect	[27,50]
		ischemia-reperfusion damage	not analyzed	[33]
	*Popdc2*^-/-^	Stress-induced sinus bradycardia	not analyzed	[27]
**zebrafish**	*popdc1* morphants	AV-block, pericardial effusion	muscular dystrophy	[28]
	*popdc2* morphants	AV-block, pericardial effusion	muscular dystrophy	[53]
	*popdc1*^S191F^	AV-block, pericardial effusion	muscular dystrophy	[28]
**human**	*POPDC1^S201F^*	2nd degree AV-block	limb-girdle MD	[28]
	*POPDC1*^del56 V217-K272^	1st degree AV-block,	limb-girdle MD	[54]
	POPDC2^W188X^	3rd degree AV-block	no known phenotype	[55]
